# Effect of Prickly Pear Cactus Peel Supplementation on Milk Production, Nutrient Digestibility and Rumen Fermentation of Sheep and the Maternal Effects on Growth and Physiological Performance of Suckling Offspring

**DOI:** 10.3390/ani10091476

**Published:** 2020-08-22

**Authors:** Sabrin A. Morshedy, Aymen E. Abdal Mohsen, Mohamed M. Basyony, Rafa Almeer, Mohamed M. Abdel-Daim, Yassmine M. El-Gindy

**Affiliations:** 1 Fish and Animal Production Department, Faculty of Agriculture (Saba Basha), Alexandria University, P.O. Box 21531, Alexandria 21500, Egypt; y.m.elgindy@alexu.edu.eg; 2 Animal Production Research Institute, Agriculture Research Center, Doki 12622, Egypt; aymanan19@hotmail.com (A.E.A.M.); mohamed000basyony@yahoo.com (M.M.B.); 3 Department of Zoology, College of Science, King Saud University, P.O. Box 2455, Riyadh 11451, Saudi Arabia; ralmeer@ksu.edu.sa (R.A.); abdeldaim.m@vet.suez.edu.eg (M.M.A.-D.); 4 Pharmacology Department, Faculty of Veterinary Medicine, Suez Canal University, Ismailia 41522, Egypt

**Keywords:** prickly pear cactus peels, Barki ewes, nutrient digestibility, rumen fermentation, maternal effect, physiological status

## Abstract

**Simple Summary:**

The agricultural byproducts commonly used as a feedstuff depend on their high abundance and the nutritional composition. Moreover, several agricultural byproducts are a valuable source of active ingredients, which could be used as feed supplements to improve the quality of formulated diets and animal performance. The present study evaluated the use of prickly pear cactus peels (*Opuntia ficus*-*indica,* PPCP) as an agricultural byproduct rich with phytochemicals in the diet of lactating Barki ewes at two doses (5 and 10 g/head/day). Supplementation with 5 g PPCP improves the milk production and composition of ewes. The maternal effect of supplementation of both doses of PPCP in the diets of lactating Barki ewes has been successful to improve the serum lipid profile, kidney function, hormonal and antioxidant status of their suckling lambs. Moreover, supplementation with 5 g PPCP improves the nutrient digestibility and rumen fermentation parameters in the sheep.

**Abstract:**

Prickly pear cactus peels (*Opuntia ficus*-*indica*, PPCP) are sustainable byproducts available in arid regions and a rich source of antioxidants. Fifteen multiparous Barki ewes (2–3 years old, 46.94 ± 0.59 kg body weight, BW) at postpartum were individually distributed in three equal groups and fed diets supplemented with PPCP at doses of 0, 5 and 10 g/head/day. Lambs were individually distributed into three equal groups according to their mothers’ groups to investigate the maternal effect on lambs’ growth performance, hematology and serum metabolites. This trial lasted for 56 days from birth to weaning. Moreover, nine adult male Barki sheep with a live BW of 65.76 ± 0.54 kg were randomly allocated into three equal groups to determine the effect of PPCP on the nutrient digestibility of the experimental diets. The results indicate that supplementing PPCP at low levels (5 g/head/day) increased milk yield (*p* = 0.050), fat-corrected milk (*p* = 0.022), energy-corrected milk (*p* = 0.015) and the yield of milk constituents compared to 10 g PPCP and the control group. In addition, lambs suckling from ewes fed the diet supplemented with 5 g PPCP had a higher (*p* = 0.001) weaning BW compared to other groups. Serum total protein, globulin, superoxide dismutase, glutathione peroxidase enzyme activities and the triiodothyronine hormone improved significantly in lambs suckling from ewes fed diets supplemented with 5 g PPCP compared to the control group. Serum cholesterol profile and kidney activities were enhanced significantly in lambs suckling from ewes fed diets supplemented with 5 and 10 g of PPCP compared to the control group. The dietary supplementation of 5 g PPCP improved the crude protein digestibility, digestible crude protein value, nitrogen balance and rumen fermentation characteristics of male sheep compared to the control group. In conclusion, supplementation with 5 g PPCP improved ewes’ milk production, offspring growth and physiological status. Furthermore, it improved the crude protein digestibility and rumen fermentation characteristics of Barki sheep.

## 1. Introduction

The livestock industry is an increasingly dynamic sector that must cope with the speedily increasing demand for livestock products [1]. Lamb production has substantially risen all over the world, especially in the arid regions, to meet the needs of increasing consumption, population growth and consumer awareness in such places [1]. Moreover, the increase in animal production should be carried out in a sustainable and cleaner manner to keep this continuous growth [2]. The increase in animal protein production must be in parallel with an increase in feed production, improving feed utilization and animal health [3]. Accordingly, the usage of agricultural byproducts as feedstuffs could improve the efficiency of the value chain of both agriculture and animal production cycles [4,5].

For several decades, the agricultural byproducts commonly used as feedstuff alternatives have depended on their high volume and proximate chemical composition [6], whereas the inclusion of agro-industrial byproducts into livestock diets has involved several benefits, including lowering feeding costs, reducing the environmental impact of livestock production, increasing feed production using sustainable technologies [1]. In addition, their content of phytochemical compounds (active ingredients), especially those with antioxidant potential, could be used as feed supplements to improve the quality of formulated diets and decrease the risk of developing resistant bacteria, and enhance animal performance [4,5,7]. Moreover, using dietary supplementation with natural antioxidants is the best way to control oxidative stress, minimize free radical production, and improve animal health [8].

In addition, the need for such feed supplementation is enforced during the critical stages of the production cycle, including pregnancy, lambing, lactation and in growing young lambs, whereas oxidative stress is promoted by reactive oxygen species (ROS) accumulating at birth [9,10]. Ewes, during the transition period of milk production, especially during the early stage of lactation, have an exposed weakness in their antioxidant defense system that induces oxidative stress [11]. Moreover, high lamb mortality is witnessed in the first month of life [12], which is associated with the incidence of oxidative stress, resulting in decreased growth, increased disease susceptibility, and economic losses [13]. Consequently, proper maternal nutrition and antioxidant delivery during the early lactation period could not only improve the ewes’ health status, milk production and reduce oxidative stress, but also improve lambs’ growth and health [14,15,16].

Prickly pear cactus (*Opuntia ficus-indica*; PPC) is a rich source of new phytochemicals and pharmaceutical entities. Furthermore, PPC is well adapted in its tolerance of drought, as it is widely cultivated in semi-arid and arid regions all over the world [17]. In Egypt, approximately 58,344 tons of PPC are produced annually [18]. The peel of PPC represents about 36–48% of the whole fruit and is considered as a waste byproduct of the PPC processing industries [19].

Prickly pear cactus peels (PPCP), as an agricultural byproduct, is rich in betalain pigments, which consist a the mixture of yellow betaxanthin and red betacyanin, which are excellent radical scavengers with an antioxidant activity higher than ascorbic acid, rutin, and catechin [20]. Additionally, PPCP contains phenolic compounds [21], flavonoids such as kaempferol, quercetin, isorhamnetin and isorhamnetin glucosides [22], total carotenoids such as β-carotene [19], vitamin E and other antioxidants such sterols, esters, saponins and alkaloids [23]; it also contains a mix of mucilage and pectin [17].

Consequently, PPCP has several proven health benefits: preventing atherosclerosis, cardiac disorders, high blood pressure, decreasing the risk of diabetes, hypoglycemic, obesity, metabolic syndrome (including diabetes type 2 and obesity), avoiding non-alcoholic fatty liver disease, cerebral ischemia, rheumatism, reducing constipation, diarrhea, bloating, gastric ulcers and cancer, enhancing immunity and overall health [20,24]. Moreover, PPCP also has several biological activities, including anti-hyperlipidemic, hypercholesterolemic, anti-inflammatory, antimicrobial, and antiviral actions [24]. Commonly, the PPCP has been used as a dietary alternative feedstuff for animal feeds, including the partial replacement of grains such as corn [25,26] or barley [18]. However, the knowledge regarding the use of PPCP as a potential feed supplement in small ruminant diets due to its high content of phytochemicals is still lacking. Therefore, the objectives of the present study are to evaluate the effect of PPCP dietary supplementation on milk production and composition in lactating ewes and its maternal effect on growth performance and hematobiochemical parameters in the offspring. Moreover, the effects of PPCP supplementation on the digestibility of nutrients, nitrogen balance and ruminal fermentation parameters were investigated.

## 2. Materials and Methods

### 2.1. Plant Material Collection and Preparation

The prickly pear cactus peels (PPCP; about 3–4 mm thickness) were obtained from a commercial farm located in Nobaria area, El-Behera Governorate, Egypt. The PPCP was cut into small pieces and dried in a forced-air dry oven below 40 °C for three days, then ground well in a kitchen blender; the powder was kept at 4 °C until use. The total phenolic content of PPCP was measured by using the Folin–Ciocalteu colorimetric method [27]. The value of total phenolic compounds content, the major antioxidant component in PPCP, is 464.04 ± 8.81 eq-mg tannic acid/100 g dry matter (DM).

### 2.2. Milk Production and Composition of Ewes Fed Diet Supplemented with PPCP

#### 2.2.1. Experimental Design and Facilities

The experiment was carried out at the Experimental Station of Nobaria, Animal Production Research Institute, Agriculture Research Center, Doki, Egypt. This study was carried out during the Egyptian autumn (March–June 2019). Fifteen healthy Barki ewes (2–3 years old, 2nd or 3rd parities), with a live BW of 46.94 ± 0.59 kg after parturition were individually distributed into three equal groups in a completely random design (5 ewes each). The control group were fed the basal diet without any supplementation (Table 1). The other two groups were fed the basal diet supplemented with 5 and 10 g PPCP/head/day. The PPCP powder was added daily to the concentrate mixture concerning the treatment. The ewes were individually housed in semi-open sheds with sand floors bedded with rice straws at a density of about 4.5 m^2^ per ewe. The experiment lasted for 56 days from parturition to the weaning of their lambs.

The minimum number of lactating ewes necessary to test the hypotheses without affecting the statistical power, and to fulfill the 3Rs protocol, were used [28,29]. The care and management of animals and the blood sample collection procedures were approved by the Ethical Committee of Alexandria University, Egypt (Approval No. AU: 14/20/04/13/3/04).

#### 2.2.2. Milk Yield and Composition of Ewes

Milk yield (MY) was recorded at 8:00 a.m. by hand milking. The milking process was performed by trained personnel until a complete udder emptying, where the lambs were separated from their mothers 12 h before the hand milking. MY was calculated over a 24-h period. The MY for individual ewes was recorded once weekly following lambing using a digital electronic scale. Milk samples (50 mL) were collected once weekly in sterilized plastic bottles and analyzed immediately for total solids, fat, protein and ash by using a Milko Scan FT 6000, Foss Electric, Hillerod, Denmark. Lactose was calculated by the following equation: total solids—(fat + protein + ash). Solids-not-fat were obtained by subtracting the fat content from the total solids of milk.

Moreover, fat-corrected milk (FCM), based on 4% fat [30] energy-corrected milk (ECM) [31] was calculated according to the following equations:

FCM (kg/d) = 0.4 × MY, g/day + 15.0 × fat yield, g/day(1)

ECM (g/day) = MY, g/day × ((0.0929 × milk fat, %) + (0.0563 × milk protein, %) + (0.0395 × milk lactose, %))/0.749 (constant for net energy for lactation, Mcal/kg)(2)

### 2.3. Maternal Effect of Dietary Supplementation of PPCP on Suckling Offspring

#### 2.3.1. Growth Performance of Lambs

Lambs were individually distributed into three equal groups (5 lambs each) according to their mother groups to investigate the maternal effect during lactation on growth performance, hematology and the serum metabolites of lambs suckling from ewes fed diets supplemented with PPCP at doses 0, 5 and 10 g/day. This trial lasted for 56 days from birth to weaning. Throughout the experiment, lambs were allowed to stay with their mothers from 08:00 a.m. to 18:00 p.m., except for milk production estimation days, when the lambs were allowed to stay with their mothers from 8:00 a.m. to 20:00 p.m.

From the third week, lambs were fed a supplementary diet containing Egyptian clover hay and the concentrate mixture in small quantities, with a protein content of 18–20%. The concentrate mixture was composed of soybean meal—15%, yellow corn—65%, wheat bran—18%, salt—0.5%, limestone—1.4%, and mineral salts—0.1%.

Lambs were tagged with ear numbers, then weighed at birth and weaning after 8 h of fasting. The average daily gain (ADG) was calculated as the body weight gain divided by the number of experimental days.

#### 2.3.2. Hematological Parameters of Lambs

Two blood samples (5 mL each) were collected at the end of the experiment. The blood samples were collected via jugular vein puncture using a gauge needle (size 20) after clipping and sterilizing the area of collection. The first blood sample was collected in a clean, dry vacuum tube with an anticoagulant (ethylenediaminetetraacetic acid; EDTA) to determine the hematological parameters.

Red blood cells (RBCs) and white blood cell (WBCs) were manually counted using a standard Neubauer cell counting chamber. RBCs were counted after diluting blood samples 200 times with a diluting fluid (10% sodium sulphate, 2% sodium chloride and mercuric chloride 1% solution), while WBCs were counted after diluting blood samples 20 times with a diluting fluid (1.5% glacial acetic acid solution and a few crystals of gentian violet). Packed cell volume (PCV) was manually measured after centrifugation at 750× *g* for 20 min. Hemoglobin concentration (Hb) was determined colorimetrically using commercial kits (Biodiagnostic Co., Cairo, Egypt) according to the cyanmethemoglobin procedure [32].

#### 2.3.3. Serum Biochemical Parameters of Lambs

The second blood sample was collected without anticoagulant in plain vacuum tubes. the samples were centrifuged (700× *g*, 15 min.) and the serum was carefully decanted into serum vials and stored at −20 °C until use for biochemical analysis. The serum glucose, total protein, albumin, cholesterol, triglycerides and high-density lipoprotein–cholesterol (HDL-c) were determined colorimetrically by using commercial kits produced by Biodiagnostic Co., Cairo, Egypt. Serum globulin level was calculated by subtracting the serum albumin from serum total protein. Low-density lipoprotein–cholesterol (LDL-c) was calculated by the following formula:

(LDL-c = cholesterol − (HDL-c + vLDL-c)(3)

and very-low-density lipoprotein–cholesterol (vLDL-c) was calculated by dividing the values of triglycerides by a factor of 5 according to [33]. The level of serum Urea-N, creatinine and the activities of serum aspartate aminotransferase (AST), alanine aminotransferase (ALT) were also determined by commercial kits produced by Biodiagnostic Co., Cairo, Egypt.

The activities of serum superoxide dismutase (SOD), catalase (CAT), glutathione peroxidase (GPX) and the level of thiobarbituric acid-reactive substances (TBARS) were measured according to [34,35,36,37], respectively. Serum triiodothyronine (T_3_) and thyroxin (T_4_) concentrations were assessed using a commercial kit (ELISA Microplate Reader, Stat Fax 2100 model; Palm City, USA) [38].

### 2.4. Nutrient Digestibility and Rumen Fermentation of Sheep Fed Diet Supplemented with PPCP

#### 2.4.1. Nutrient Digestibility and Nitrogen Balance

Nine matured male Barki sheep with an average live BW of 65.76 ± 0.54 kg were allocated randomly into three equal groups (3 sheep each, individually housed in metabolism cages) to determine the effects of PPCP on the nutrient digestibility of the experimental diets, nutritive values (total digestible nutrients, TDN and digestible crude protein, DCP), nitrogen balance (NB) and ruminal fermentation parameters. The control group were fed a basal diet without any supplementation. The other two groups were fed the basal diet supplemented with 5 and 10 g PPCP/head/day. The PPCP powder was added daily to the concentrate mixture with respect to the treatment.

The experiment lasted for 20 days, the first 14 days were the adaptation period and the following 6 days were for collecting samples of feces, urine and refused feed before offering the morning meal. Sheep were fed Egyptian green clover (*Trifolium alexandrinum*), rice straw and the concentrate mixture with free access to clean drinking water. The diet was offered twice daily at 6:00 a.m. and 16:00 p.m., and the refusals were recorded daily to determine feed intake.

Urine excreted in 24 h was collected daily throughout the collection period in plastic buckets containing 100 mL of 10% H_2_SO_4_. Samples of 10% of the total urine were stored at −20 °C to determine NB.

Apparent digestibility coefficients for each nutrient were calculated by expressing the weight of each nutrient absorbed as a proportion of the weight consumed (nutrients consumed in feces/nutrients consumed). Digestible nutrients can be determined by multiplying digestion coefficients by the proximate analysis of feed, then calculating TDN [39] as follows:

TDN, % = DCP, % + digestible crude fiber, % + digestible nitrogen free extract, % + (digestible ether extract, % × 2.25)(4)

Digestible energy (DE) and metabolizable energy (ME) are calculated [39] as follows:

DE (kcal/kg DM) = TDN% × 44(5)

ME (kcal/kg DM) = DE (kcal/kg DM) × 0.82(6)

#### 2.4.2. Proximate Analysis of Feed and Feces

Samples of the experimental basal diet and feces were collected and dried in a forced-air oven at 60 °C for 48 h to calculate the dry matter intake. The dried samples were ground to pass a 1-mm screen and chemically analyzed for DM, crude protein (CP, as 6.25 × N), ether extract (EE) and ash [40]. The contents of neutral detergent fiber, expressed exclusive of residual ash (aNDFom), were measured using a heat-stable α-amylase and sodium sulphite [41]. Acid detergent fiber, expressed exclusive of residual ash (ADFom), was determined [42]. Lignin (sa) was determined by the solubilization of cellulose with sulfuric acid (720 mL/L) [43]. These fiber fractions were sequentially measured using an ANKOM fiber analyzer using the same sample in ANKOM filter bags and expressed exclusive of residual ash. The concentrations of hemicellulose and cellulose were estimated as the differences between aNDFom and ADFom, and between ADFom and lignin (sa), respectively. Non-fiber carbohydrates were calculated by the following equation = 1000 − (aNDFom + CP + EE + ash), and organic matter (OM) was calculated as the difference between DM and ash. Nitrogen in urine was determined by the micro-Kjeldahl method [40]. The ingredients of the concentrate mixture and chemical composition of the concentrate mixture, Egyptian green clover and rice straw are presented in Table 1.

#### 2.4.3. Rumen Fermentation Characteristics

Rumen samples (20 mL each) were collected from each adult male sheep before the morning feed at the end of the digestibility trial using a stomach tube [44]. Rumen fluid samples were filtered through four layers of cheesecloth; the pH value was measured within 2–3 min of sampling using a portable pH meter (GLP 21 model; CRISON, Barcelona, Spain). Then, the rumen samples were stored at −20 °C until future analyses. Ruminal NH_3_-N concentration was measured colorimetrically (spectrophotometer; Spectrophotometer PD-303 UV, APEL, Japan) using commercial kits (SPINREACT, Ctra. Santa Coloma, 7, Girona, Spain) as described by [45].

Individual volatile fatty acid (VFA) concentration was determined using gas chromatography (GC) with some modifications [46]. In brief, after thawing, an aliquot of 1.6 mL was prepared with 0.4 mL of 25% metaphosphoric acid (4:1 ratio) and centrifuged at 15,000× *g* for 20 min at 4 °C (K1015 Micro Prime; Centurion Scientific Ltd., Stoughton, Chichester, UK). The supernatant was used to determine the VFA concentration with a GC (Thermo Fisher Scientific, Inc., TRACE1300, Rodano, Milan, Italy) fitted with an AS3800 autosampler and equipped with a capillary column HP-FFAP (19091F-112; 0.320 mm o.d., 0.50 μm i.d., and 25 m length; J and W Agilent Technologies Inc., Palo Alto, CA, USA). Hydrogen at 1.35 mL/min was used as a carrier gas. Air, hydrogen and nitrogen fluxes (make up gas) were kept at 450, 40, and 35 mL/min, respectively. A 0.1-μL aliquot was injected in splitless mode for the entire run with 31.35 mL/min of H2 flux (63.432 Pa). Injector and flame ionization detector (FID) temperatures were isothermally held at 250 °C. The oven heating slope was 80 °C (1 min), 120 °C (20 °C/min for 3 min), and 205 °C (10 °C/min for 2 min), with a 9 min overall analysis time.

### 2.5. Statistical Analysis

Data obtained from the experiment were subjected to a one-way analysis of variance (ANOVA) using SPSS for Windows, Version 16.0. Chicago, SPSS Inc. The significance between the treatments was declared at *p* < 0.05 by applying the Duncan test [47], according to the following statistical model:

One-way model: Y_iK_ = μ + X_i_ + e_ik_ (7)

where: Y_iK_ = the response variable; μ = the overall mean; X_i_ = the fixed effect of treatment (control and 5 and 10 PPCP); e_ik_ = the residual error.

## 3. Results

### 3.1. Milk Yield and Composition of Ewes

**Figure 1:**
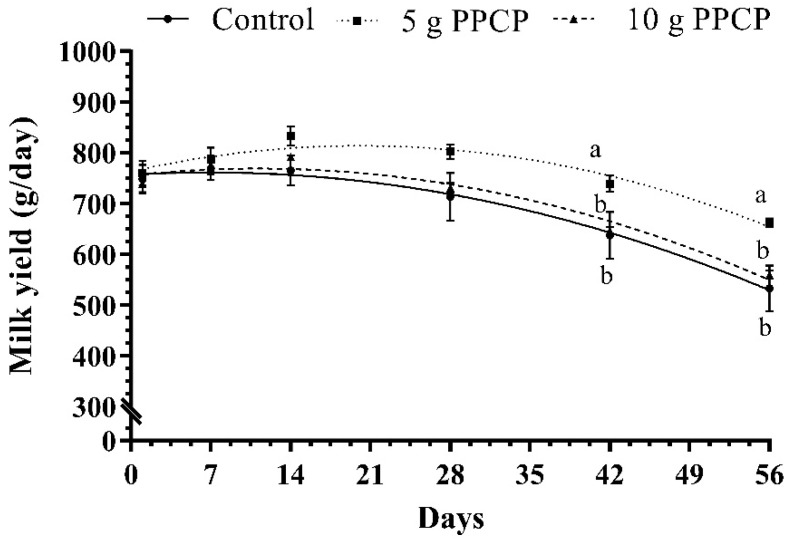
Milk yield of ewes fed with prickly pear cactus (*Opuntia ficus-indica*) peel-supplemented diets during the lactation period. ^a,b^ Means at the same sampling time marked with different superscripts are significantly different at *p* ≤ 0.05.

The effects of PCPP supplementation on MY and the yield of milk constituents are shown in Table 2 and Figure 1. The results indicate that milk fat and protein yields tended to increase with the addition of 5 g PPCP compared to the other groups. Milk ash yield increased (*p* = 0.006) with the inclusion of PPCP in a dose-dependent manner. Furthermore, supplementing PPCP at a low dose (5 g/head/day) increased MY (*p* = 0.050), milk lactose yield (*p* = 0.048), milk solids yield (*p* = 0.029), milk solids-not-fat yield (*p* = 0.047), FCM (*p* = 0.022) and ECM (*p* = 0.015) compared to the control and high PPCP dose (10 g/head/day) groups. Moreover, there are no significant differences in MY and most of the milk yield constituents between supplementing PPCP at a high dose (10 g/head/day) and the control.

### 3.2. Growth Performance of Lambs

Table 3 shows the maternal effect of dietary supplementation with PPCP on growth performance of suckling lambs during lactation and until weaning. The supplementation of 5 g PPCP in the diet of ewes improved their lambs’ weaning BW (*p* = 0.001) and ADG (*p* = 0.001) compared to the control group. However, the supplementation of PPCP at high levels reduced the lamb weaning BW and ADG compared to the control group.

### 3.3. Hematological Parameters of Lambs

The effects of maternal nutrition on the hematological parameters of lambs suckling from ewes fed diets supplemented with PPCP are illustrated in Table 4. The RBCs, WBCs, Hb and PCV of lambs suckling from ewes fed diets supplemented with PPCP were not affected.

### 3.4. Serum Biochemical Constituents of Lambs

Table 5 summarizes the effects of maternal nutrition during lactation on the serum biochemical constituents of lambs suckling from ewes fed diets supplemented with two levels of PPCP. Serum total protein and globulin improved in lambs suckling from ewes fed diets supplemented with PPCP and increased (*p* = 0.044, 0.031, respectively) with 5 g PPCP compared to the control group. Cholesterol, triglyceride, vLDL-c and LDL-c decreased significantly in lambs suckling from ewes fed diets supplemented with PPCP in a dose-dependent manner. HDL-c/LDL-c ratio was increased significantly (*p* = 0.001) in lambs suckling from ewes fed the diet supplemented with PPCP compared to the control group. Serum urea and creatinine levels were lowered significantly (*p* = 0.001, 0.013) in lambs suckling from ewes fed diets supplemented with PPCP compared to the control group. Serum glucose, HDL-c, AST and ALT did not differ significantly among different treatments.

### 3.5. Antioxidant Status and Thyroid Gland Hormone of Lambs

**Figure 2:**
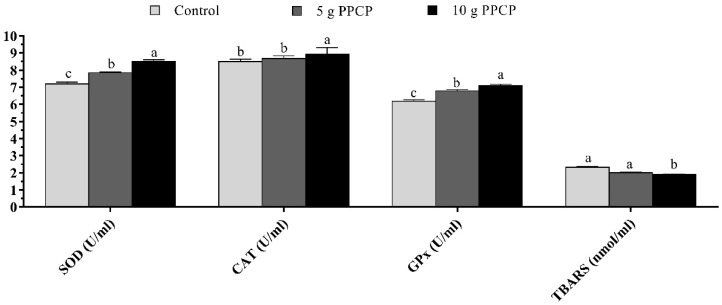
Antioxidant status of lambs suckling from ewes fed diets supplemented with prickly pear cactus (*Opuntia ficus*-indica) peels until weaning (56 days). ^a,b,c^ Columns bearing with different superscript are significantly different (*p* < 0.05). Superoxide dismutase (SOD); catalase (CAT); glutathione peroxidase (GPX); thiobarbituric acid-reactive substances (TBARS).

Serum SOD and GPX activities increased significantly (*p* = 0.001) in lambs suckling from ewes fed diets supplemented with PPCP at both doses compared to the control group (Figure 2). Serum CAT activity increased significantly (*p* = 0.017) in lambs suckling from ewes fed the diet supplemented with 10 g PPCP compared to 5 g PPCP and control groups. There was a significant decrease (*p* = 0.002) in TBARS value in lambs suckling from ewes fed the diet supplemented with 10 g PPCP compared to 5 g PPCP and control groups. However, 5 g PPCP treatment tended to increase the serum CAT activity and decrease the serum TBARS value of lambs compared to the control group.

**Figure 3:**
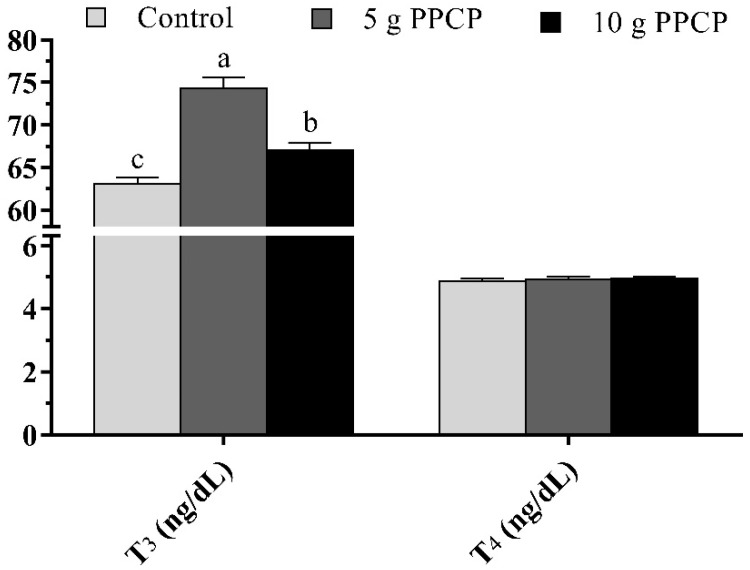
Serum metabolic hormones level (triiodothyronine (T_3_) and thyroxin (T_4_)) of lambs suckling from ewes fed diets supplemented with prickly pear cactus (*Opuntia ficus*-*indica*) peels until weaning (56 days). ^a,b,c^ Columns bearing with different superscript are significantly different (*p* < 0.05).

Serum metabolic hormones (T_3_ and T_4_) of lambs suckling from ewes fed diets supplemented with PPCP during the lactation period until weaning are shown in Figure 3. The T_3_ hormone was significantly higher (*p* = 0.001) in lambs suckling from ewes fed diets supplemented with PPCP at 5 and 10 g/head/day compared to the control group and the highest value was recorded with 5 g PPCP. However, the T_4_ hormone was not affected by PPCP treatments.

### 3.6. Nutrient Digestibility and Nitrogen Balance of Sheep

The nutrient digestibility and nitrogen balance of sheep fed diets supplemented with PPCP are presented in Table 6. The dietary supplementation of 5 g PPCP significantly increased the digestibility of CP, DCP and NB; however, nitrogen intake and nitrogen absorption tended to improve with the addition of 5 g PPCP in the diet compared to the control group. Fiber fraction digestibility increased significantly due to the dietary supplementation of 5 g PPCP, whereas CF tended to improve by PPCP supplementation at the lower dose compared to the control group. Moreover, the digestibility of DM, OM, EE and NFE, TDN, DE and ME tended to improve due to the dietary supplementation of 5 g PPCP compared to the control group. On the other hand, the dietary supplementation of 10 g PPCP decreased all digestibility trial criteria. In addition, there were no significant differences in the total dry matter intake of sheep between 5 g PPCP and control groups; meanwhile, it decreased with supplementation of 10 g PPCP.

### 3.7. Rumen Fermentation Characteristics of Sheep

The rumen fermentation of sheep fed diets supplemented with PPCP after the digestibility trial is illustrated in Table 7. The NH_3_-N concentrations decreased significantly (*p* = 0.001) due to the dietary supplementation of PPCP compared to the control group. Total volatile fatty acids and acetic acid concentrations increased significantly due to the dietary supplementation of 5 g PPCP. Propionate value decreased significantly due to the dietary supplementation of 10 g PPCP compared to other groups. The dietary supplementation of PPCP significantly increased butyrate concentration in a dose-dependent manner compared to the control group. Ruminal fluid pH was similar in sheep fed diets supplemented with PPCP and the control group.

## 4. Discussion

Noteworthily, nutrition during lactation is critical for milk production [48], whereas milk production is an energy demand process which causes stress in the animal, especially during the early stage of lactation due to the associated extreme physiological and metabolic changes [8,49]. Accordingly, introducing complete balanced diets alone as an animal requirement in this stage of production is not good enough, but also using supplemented diets with natural antioxidant is an important measure of animal husbandry, especially under controlled farming systems, to assimilate natural pasture-living animals [4,15]. PPCP is a waste material of the PPC processing industries and represents about 36–48% of the whole fruit [19]. It is considered an excellent source of bioactive compounds and pharmaceutical substances [23].

In the current findings, the dietary supplementation of 5 g PPCP directly after lambing significantly improved Barki ewes’ milk production and milk yield constituents. This increase in milk production could be associated to the antioxidant components of PPCP such as phenols, tannins, as well as betalains [50], which have been reported to improve mammary secretory cell health, secretory activity and the ejection ability of alveoli linked positively to MY [51]. Additionally, the increase in MY might be due the increase in serum prolactin hormone, as indicated by the dietary supplementation of 2, 10, and 70 mg/kg of *V. agnus-castus* leaf extract [52]. Moreover, phytochemical supplementation stimulates the secretion of some hormones, such as growth hormone, that promote milk production during the lactation period by increasing the rate of change of nutrients from the body to the mammary gland, as well as glucocorticoid hormones that play a vital role in the initiation and maintenance of lactation [53]. Furthermore, the effects of the polyphenolic compound on milk production might be due to an improvement in rumen fermentation in the current study rather than an altered metabolism [54]. Moreover, there were no significant differences in MY and most of the milk constituents yielded between the supplementing PPCP at high dose (10 g/head/day) and the control group. This effect could be due to the decrease in the feed intake of ewes fed diets supplemented with a higher level of PPCP, as indicated in El-Gindy et al. (unpublished data) and the digestibility trial in the present study, which could be due to its high content of organic acids compared to other genotypes, which results in an acidic smell and negatively affects the feed intake [55]. In addition, agro-industrial byproducts often contain anti-nutritive compounds, which may lower both their palatability and digestibility, thus influencing the feed utilization, health and production of animals [56]. PPCP has also been reported to contain some anti-nutritive compounds, such as tannins, condensed tannins, phytate and oxalate, and high levels of them could have undesirable effects on feed intake and animal production [57].

Moreover, supplementing PPCP at a low dose (5 g/head/day) increased the milk lactose yield (*p* = 0.048) compared to the control and high PPCP dose (10 g/head/day) groups. In accordance with the present results, dietary supplementation with prickly pear, *Nopalea cochenillifera,* increased the milk lactose of Saanen goats [58]. This increase in milk lactose could be due to the antioxidant components of PPCP such as phenols [59]. The improvement in fat yield and FCM may be due to the fact that PPCP is characterized by high antioxidant contents such as phenolic compounds that are higher in the peel than the pulp [4], which are associated with the capability of polyphenols to modify the biohydrogenation of fatty acids in the rumen [60], thus causing an improvement in the lipid fraction quality of the dairy products [61]. Additionally, increasing the FCM by dietary supplementation with PPCP may be related to the increase in total VFA and acetate concentrations in the current results and supported by the previous results [62]. In contrast, the use of PPC cultivars as forage did not affect FCM [63]. This could be due to the differences in the active components of cactus leaves (PPC) and fruit peel (PPCP), as used in the current study.

The increase in milk protein yield with PPCP supplementation could be due to the fact that PPCP contains tannins, which reduce the ruminal degradation of CP from the diet, leading to an increasing amount of protein being available in the small intestine [4,64,65]. Moreover, the increase in ECM in the present study may be attributed to the flavonoid content in PPCP. The data obtained are consistent with [54] indicated that ECM increased in the cows supplemented with a plant extract rich in flavonoids. Accordingly, the phytochemical constituents of PPCP play a key role in its milk production stimulating effect via the interaction with the physiological processes related to milk production, including an improvement in antioxidant status (El-Gindy et al., unpublished data) and hormonal regulation.

Maternal effects in sheep may be more important than in cattle or swine because of the higher relative variation in litter size and the dependence of many lambs on their mother’s milk supply until weaning [66]. The present results indicated that the supplementation of 5 g PPCP in the diet of ewes improved their lambs’ weaning BW and ADG compared to the control group. The current results agree with results from another study showing that lambs from *Opuntia*-fed ewes grew faster (*p* < 0.01) and had heavier BWs at weaning (*p* < 0.05) than lambs from the other groups [67]. The better growth of lambs suckling from ewes fed diets supplemented with 5 g PPCP compared to lambs fed the 10 g PPCP treatment in the current study could be mainly due to the increase in the milk production of the treated ewes (Table 2 and Figure 1), particularly in the last 2 weeks before weaning (42–56 days). The obtained results agreed with those of David et al. [68] who found that maternal effects depend on the mother’s ability to produce the milk required for lambs’ growth. Furthermore, the greater growth of lambs could be associated to the increase in the percentage and yield of milk fat and protein in the current results, which are considered important factors for lambs’ growth and survival rate [69]. Furthermore, the better growth in the current study may be attributed to polyphenols and other phytochemicals in PPCP which could transfer to the milk of ewes and consequently to their offspring. In agreement, the dietary polyphenols during the lactation lead to an enhancement in the metabolic reprogramming of the offspring [15,16]. Moreover, the better growth in the current study may be attributed to the enhancement of serum antioxidant status and the T_3_ hormone of lambs suckling from ewes fed diets supplemented with PPCP (Figure 2 and Figure 3).

Regarding the hematobiochemical investigation of the offspring, the maternal effect of dietary supplementation of PPCP did not affect the hematological parameters of lambs. Meanwhile, cholesterol, triglyceride, vLDL-c and LDL-c decreased significantly in lambs suckling from ewes fed diets supplemented with PPCP compared to the control group. This decrease could be ascribed to reducing the cholesterol synthesis and its absorption, which resulted from the functional effects of phenolic and polyphenolic antioxidants [70]. Polyphenol supplementation markedly decreased the blood levels of triacylglycerols and cholesterol [50]. Furthermore, PPCP contains flavonoids and betalains, which have a hypolipidemic activity [71].

In addition, pectin in PPCP could inhibit cholesterol and lipid synthesis by promoting the conversion of cholesterol to bile acids [72,73], by stimulating the catabolism of cholesterol [72], by changing hepatic cholesterol metabolism without affecting cholesterol absorption [74], or by lowering lipid peroxidation by increasing antioxidant enzymes [75] including SOD, CAT and GPX, as indicated in the current study. Likewise, glycoprotein isolated from *Opuntia* varieties used in folk medicine has potent antioxidant and hypolipidemic properties in mice, thus inhibiting lipoprotein lipase [75].

Serum levels of AST, ALT and the ratios between them were not affected in lambs suckling from ewes fed diets supplemented with PPCP, without any side effects on the metabolic activity of hepatic tissue. The obtained findings are in harmony with El-Neney et al. [76], who found that AST and ALT activities were not affected by dietary treatments supplemented with PPCP. Moreover, lambs suckling from ewes fed diets supplemented with PPCP had lower (*p* = 0.001, 0.013) serum urea-N and creatinine compared to the untreated group, which led to improved renal function in the present study, which was clearly evident in the low blood urea and creatinine. The significant decrease in urea-N and creatinine due to treatment with PPCP could be associated with the direct modification of rumen fermentation and ammonia production in the present study which concurs with [77].

Furthermore, pathogenesis and other forms of oxidative stress are promoted by ROS accumulating at birth in the mother and offspring, if antioxidants are inadequate [9,10]. In the offspring, the high growth rate resulted in a high production of ROS by metabolic processes and the induction of oxidative stress, which could increase mortality rates and decrease the growth of lambs, thus resulting in economic losses [13]. Noteworthily, the present results showed that lambs suckling from ewes fed diets supplemented with PPCP at both doses succeed to avoid such oxidative stress through a significant increase in serum SOD and GPX activities. However, the antioxidant values in the 5 g/head/day supplemented group are lower than 10 g/head/day; this could be due to the fast growth of this group, which could increase the production of ROS, which, in turn, depletes antioxidant enzymes [13]. The positive effect of PPCP on blood antioxidant levels is in agreement with López-Romero et al. [78]. The antioxidant mechanism of PPCP could be due to the high content of polyphenols and other phytochemical constituents that are absorbed in the small intestine [79]. The vertical transmission of polyphenols and PPCP active components via milk to the offspring was confirmed [16,80]. These compounds have antioxidant properties [21,74,78], which could modulate the antioxidant balance in the present study. Moreover, the current findings indicated that dietary supplementation of 5 g PPCP improved the nutrient digestibility, nutritive values, DE and NB in sheep compared to the control group. In parallel with the present findings, the increase in DM, OM and CP total and ruminal digestibility induced by the spineless cactus replacing Tifton hay in sheep could be interpreted by the availability and better use of nutrients for ruminal microorganisms [81]. Furthermore, the digestibility of CP, total carbohydrates, and TDN increased (*p* < 0.05) in lambs fed spineless cacti [82]. According to Siqueira et al. [81], spineless cacti consist of non-fibrous carbohydrates that represent the largest fraction of the total carbohydrates, and are readily fermentable in the rumen. Nevertheless, the current results disagreed with Inácio et al. [83], who found that the digestibility of OM, CP and NDF, and NB did not change between the control diet and cactus *Opuntia*-based diets. Meanwhile, there was a decrease in the feed intake and nutrient digestibility of sheep fed diets supplemented with a higher level of PPCP in the current findings, which could be due to its content of organic acids being higher than other genotypes, resulting in the acidic smell, and also the presence of anti-nutritive compounds in PPCP such as tannins, condensed tannins, phytate and oxalate, which could negatively affect the feed intake and nutrient digestibility, especially in high levels of PPCP [55,56]. The current findings disagree with [84], who found that the dry matter intake increased by increasing the levels of PPC in the diet of dairy goats.

An analysis of the ruminal constituents in sheep fed PPCP-supplemented diets in the current study revealed that the dietary supplementation of PPCP leads to a better utilization of protein by decreasing the ruminal NH_3_-N concentration compared to the control group. Additionally, the dietary supplementation of 5 g PPCP significantly increased CP, DCP and NB. Similarly, the ruminal NH_3_-N concentration was reduced (*p* < 0.01) in sheep fed diets supplemented with *Opuntia* [85]. In the same sense, the addition of tannins and polyphenols in the diets of ruminants at moderate levels can improve the performance of animals due to the better utilization of protein intake [56,64]. This occurs throughout binding proteins, consequently lowering their ruminal degradation and resulting in a high flow of amino acids to the small intestine [4,65].

The dietary supplementation of PPCP improved the rumen fermentation characteristics of sheep by increasing the total VFA concentration and VFA (acetate and butyrate) proportions compared to the control group. This increase in VFA may be due to the higher content of PPC in easily fermented carbohydrates [86]. The observed increase in total VFA and acetate concentrations may be due to increased cellulolytic bacteria activity in the rumen [87]. Moreover, the reduction in ruminal NH_3_-N concentration in sheep fed diets supplemented with PPCP correlates with the higher ruminal total VFA, indicating an increase in fermentation rate and higher microbial protein synthesis [88]. However, other studies reported lower total VFA, acetate and butyrate concentrations with no alteration in the propionate concentration in sheep fed diets supplemented with *Opuntia* [85] or no change in VFA proportions following the addition of spineless cactus as a replacement for Tifton hay [81].

## 5. Conclusions

In light of the above findings, the lower level of prickly pear cactus peel (5 g/head/day) supplementation improved lactating Barki ewes’ productive performance and growth, and the physiological status of their offspring. Furthermore, it enhanced their feed utilization via improving the digestibility and rumen fermentation parameters of the sheep. Therefore, prickly pear cactus peels could be an agricultural byproduct with a potential use as a cheap and available feed supplement in the diet of lactating Barki ewes reared in arid regions for more sustainable animal production.

## Figures and Tables

**Table 1:** Ingredients of the concentrate mixture and chemical composition of concentrate mixture, Egyptian clover (*Trifolium alexandrinum*) and rice straw used in the present study.

**Ingredients of the Concentrate Mixture**		**g/kg DM**	
Yellow corn		300.00	
Soybean meal		120.00	
Wheat bran		100.00	
Barley		300.00	
Sugar beet pulp		100.00	
Molasses		50.00	
Salt		10.00	
Limestone		15.00	
Premix ^1^		5.00	
**Chemical Composition, g/kg DM**	**Concentrate Mixture**	**Egyptian Clover**	**Rice Straw**
Dry matter	894.40	208.60	881.20
Organic matter	937.20	936.70	926.70
Crude protein	152.60	116.30	32.70
Ether extract	29.60	19.80	16.20
Ash	62.80	63.30	73.30
ANDFom ^2^	364.40	598.20	668.30
ADFom ^3^	258.90	416.30	447.30
Lignin (sa)	58.60	82.70	104.10
Non-fiber carbohydrates ^4^	390.60	202.40	209.50
Hemicellulose ^5^	105.50	181.90	221.00
Cellulose ^6^	200.30	333.60	343.20

^1^ Premix (mg/Kg): Zn, 60 mg; Mn, 80 mg; Fe, 35 mg; Cu, 8 mg; Se, 0.6 mg; vitamin D3, 2500 ICU; vitamin A, 12,000 IU; vitamin E, 20 IU; menadione, 1.3 mg; riboflavin, 5.5 mg; vitamin B12, 10 μg; vitamin B6, 3 mg; thiamine, 3 mg; folic acid, 1.0 mg; d-biotin, 50 μg; Ca-pantothenate, 1 mg; nicotinic acid, 50 mg and choline chloride, 600 mg.^2^ Neutral detergent fiber expressed exclusive of residual ash (aNDFom); ^3^ acid detergent fiber expressed exclusive of residual ash (ADFom); ^4^ non-fiber carbohydrates = 1000 − (aNDFom + crude protein + ether extract + ash); ^5^ Hemicellulose = aNDFom − ADFom; ^6^ cellulose = ADFom − Lignin (sa).

**Table 2:** Milk yield and yield of milk components of ewes fed diets supplemented with prickly pear cactus (*Opuntia ficus-indica*) peels during the lactation period.

Items		Prickly Pear Level (g/head/day)			SEM	*p* Value
		0	5	10		
Milk yield, g/day		694.17 ^b^	763.83 ^a^	699.33 ^b^	14.264	0.050
Milk Components, g/day	Fat	22.93 ^ab^	24.97 ^a^	21.25 ^b^	0.568	0.013
	Protein	23.13 ^a^	24.05 ^a^	19.52 ^b^	0.669	0.003
	Lactose	40.12 ^b^	46.92 ^a^	44.47 ^ab^	1.194	0.048
	Ash	5.37 ^b^	6.39 ^a^	6.51 ^a^	0.179	0.006
	Solids	91.56 ^b^	102.34 ^a^	91.75 ^b^	2.015	0.029
	Solids-not-fat	68.63 ^b^	77.36 ^a^	70.50 ^ab^	1.595	0.047
	FCM ^1^	621.66 ^b^	680.12 ^a^	598.50 ^b^	13.360	0.022
	ECM ^2^	669.90 ^b^	738.00 ^a^	644.82 ^b^	14.839	0.015

^a,b^ means with a different superscript in the same row are significantly different (*p* < 0.05).^1^ fat-corrected milk (FCM), g/day; ^2^ energy-corrected milk (ECM), g/day. Milk yield and yield of milk components, g/day.

**Table 3:** Birth and weaning body weight, and average daily gain of lambs suckling from ewes fed diets supplemented with prickly pear cactus (*Opuntia ficus*-*indica*) peels.

Items	Prickly Pear Level (g/head/day)			SEM	*p* Value
	0	5	10		
Birth bodyweight, kg	3.08	3.11	3.09	0.035	0.946
Weaning bodyweight, kg	16.79 ^b^	18.02 ^a^	14.80 ^c^	0.408	0.001
Average daily gain, kg/day	0.230 ^b^	0.250 ^a^	0.196 ^c^	0.007	0.001

^a,b,c^ Means with a different superscript in the same row are significantly different (*p* < 0.05).

**Table 4:** Hematological parameters of lambs suckling from ewes fed diets supplemented with prickly pear cactus (*Opuntia ficus*-*indica*) peels.

Items	Prickly Pear Level (g/head/day)			SEM	*p* Value
	0	5	10		
RBCs (×10^6^/mm^3^) ^1^	9.19	9.93	10.86	0.489	0.430
Hb (g/dL) ^2^	11.69	11.82	12.10	0.322	0.900
WBCs (×10^3^/mm^3^) ^3^	9.35	9.48	10.08	0.347	0.716
PCV (%) ^4^	38.10	39.30	36.79	0.542	0.168

^1^ Red blood cell (RBC) counts; ^2^ hemoglobulin (Hb); ^3^ white blood cell (WBC) counts; ^4^ packed cell volume (PCV).

**Table 5:** Serum biochemical constituents of lambs suckling from ewes fed diets supplemented with prickly pear cactus (*Opuntia ficus*-*indica*) peels.

Items	Prickly Pear Level (g/head/day)			SEM	*p* Value
	0	5	10		
**Glucose and Protein Profile**					
Glucose, mg/dL	53.92	53.49	54.07	0.161	0.346
Protein, g/dL	6.95 ^b^	7.35 ^a^	7.11 ^ab^	0.072	0.044
Albumin, g/dL	3.09	3.21	3.15	0.032	0.184
Globulin, g/dL	3.86 ^b^	4.13 ^a^	3.96 ^ab^	0.050	0.031
Albumin/Globulin ratio	0.80	0.78	0.80	0.008	0.283
**Lipid Profile ^1^**					
Cholesterol, mg/dL	60.53 ^a^	57.66 ^b^	56.39 ^b^	0.663	0.003
Triglyceride, mg/dL	41.44 ^a^	39.54 ^b^	37.60 ^c^	0.571	0.0001
HDL-c, mg/dL	27.59	27.71	28.15	0.244	0.671
vLDL-c, mg/dL	8.29 ^a^	7.91 ^b^	7.52 ^c^	0.114	0.0001
LDL-c, mg/dL	24.54 ^a^	22.16 ^b^	20.72 ^c^	0.569	0.0001
HDL-c/LDL-c ratio	1.13 ^c^	1.25 ^b^	1.36 ^a^	0.023	0.001
**Liver and Kidney Function ^2^**					
Urea, mg/dL	39.42 ^a^	35.42 ^b^	33.99 ^c^	0.820	0.0001
Creatinine, mg/dL	0.82 ^a^	0.77 ^b^	0.75 ^b^	0.011	0.013
AST, U/L	30.10	30.60	29.98	0.191	0.431
ALT, U/L	17.57	18.08	17.21	0.165	0.072
AST/ALT ratio	1.71	1.69	1.74	0.016	0.534

^a,b,c^ Means with a different superscript in the same row are significantly different (*p* < 0.05).^1^ High-density lipoprotein–cholesterol (HDL-c), low-density lipoprotein–cholesterol (LDL-c, mg/dL) = cholesterol, mg/dL -HDL-c, mg/dL-very low-density lipoprotein–cholesterol (vLDL-c), mg/dL, very low-density lipoprotein–cholesterol (vLDL-c, mg/dL) = triglyceride, mg/dL/5; ^2^ aspartate aminotransferase (AST), alanine aminotransferase (ALT).

**Table 6:** Total feed intake, digestibility coefficient, nutritive value and nitrogen balance of diets supplemented with prickly pear cactus (*Opuntia ficus*-*indica*) peels in adult male sheep.

Items	Prickly Pear Level (g/head/day)			SEM	*p* Value
	0	5	10		
Total Dry Matter Intake, g/d	1585.39 ^a^	1585.06 ^a^	1487.44 ^b^	18.870	0.016
**Digestible Coefficient, %**					
Dry matter	63.18 ^a^	64.47 ^a^	57.44 ^b^	1.117	0.001
Organic matter	67.55 ^a^	68.46 ^a^	62.33 ^b^	0.988	0.001
Crude protein	59.87 ^b^	63.00 ^a^	56.84 ^c^	0.958	0.003
Crude fiber	57.77 ^a^	60.50 ^a^	51.64 ^b^	1.382	0.001
Ether extract	76.98 ^a^	78.39 ^a^	68.93 ^b^	1.512	0.001
Nitrogen free extract	72.74 ^a^	73.01 ^a^	67.38 ^b^	0.936	0.001
ANDFom ^1^	56.67 ^b^	58.64 ^a^	52.71 ^c^	0.901	0.001
ADFom ^2^ appropriate	53.85 ^b^	55.64 ^a^	47.69 ^c^	1.210	0.001
Lignin (sa)	43.75 ^b^	45.15 ^a^	36.74 ^c^	1.302	0.001
**Nutritive Value, %**					
TDN ^3^	63.99 ^a^	64.87 ^a^	59.25 ^b^	0.900	0.001
DCP ^4^	6.68 ^b^	7.04 ^a^	6.53 ^b^	0.084	0.008
DE ^5^, kcal/kg	2815.56 ^a^	2854.13 ^a^	2607.15 ^b^	39.608	0.001
ME ^6^, kcal/kg	2308.76 ^a^	2340.39 ^a^	2137.86 ^b^	32.478	0.001
**Nitrogen Balance, g/d**					
Nitrogen intake	28.29 ^a^	28.32 ^a^	27.35 ^b^	0.185	0.018
Nitrogen absorbance	16.94 ^a^	17.84 ^a^	15.55 ^b^	0.359	0.003
Nitrogen balance	4.92 ^b^	6.58 ^a^	3.96 ^b^	0.414	0.003

^a,b,c^ Means with a different superscript in the same row are significantly different (*p* < 0.05).^1^ aNDFom: neutral detergent fiber expressed exclusive of residual ash; ^2^ acid detergent fiber expressed exclusive of residual ash (ADFom); ^3^ total digestible nutrients (TDN); ^4^ digestible crude protein (DCP); ^5^ digestible energy (DE); ^6^ metabolizable energy (ME).

**Table 7:** Rumen fermentation parameters in adult male sheep fed diets supplemented with prickly pear cactus (*Opuntia ficus*-*indica*) peels.

Items	Prickly Pear Level (g/head/day)			SEM	*p* Value
	0	5	10		
Rumen pH	6.30	6.28	6.24	0.021	0.602
NH_3_-N (mg/100 mL)	13.69 ^a^	12.47 ^b^	11.75 ^c^	0.297	0.001
Total volatile fatty acids (meq/100 mL)	11.16 ^b^	12.66 ^a^	12.19 ^ab^	0.272	0.038
Acetate (mol/100 mol)	54.31 ^b^	57.35 ^a^	57.20 ^ab^	0.575	0.017
Propionate (mol/100 mol)	25.00 ^a^	24.37 ^a^	23.19 ^b^	0.314	0.023
Butyrate (mol/100 mol)	7.73 ^b^	8.91 ^a^	9.01 ^a^	0.234	0.012

^a,b,c^ Means with a different superscript in the same row are significantly different (p < 0.05).

## References

[1] Berbel J., Posadillo A. (2018). Review and analysis of alternatives for the valorisation of agro-industrial olive oil by-products. Sustainability.

[2] McCoard S.A., Stevens D.R., Whitney T.R. (2020). Sustainable sheep and goat production through strategic nutritional management and advanced technologies. Animal Agriculture.

[3] Ward M., Gifford C. (2017). Sheep Nutrition.

[4] Correddu F., Lunesu M.F., Buffa G., Atzori A.S., Nudda A., Battacone G., Pulina G. (2020). Can agro-industrial by-products rich in polyphenols be advantageously used in the feeding and nutrition of dairy small ruminants?. Animals.

[5] Lai W.T., Khong N.M., Lim S.S., Hee Y.Y., Sim B.I., Lau K.Y., Lai O.M. (2017). A review: Modified agricultural by-products for the development and fortification of food products and nutraceuticals. Trends Food Sci. Technol..

[6] Volpe M., Goldfarb J.L., Fiori L. (2018). Hydrothermal carbonization of *Opuntia ficus-indica* cladodes: Role of process parameters on hydrochar properties. Bioresour. Technol..

[7] Gómez-García R., Campos D.A., Aguilar C.N., Madureira A.R., Pintado M. (2020). Valorization of melon fruit (*Cucumis melo* L.) by-products: Phytochemical and biofunctional properties with emphasis on recent trends and advances. Trends Food Sci. Technol..

[8] Abuelo A., Hernández J., Benedito J.L., Castillo C. (2019). Redox biology in transition periods of dairy cattle: Role in the health of periparturient and neonatal animals. Antioxidants.

[9] Gruse J., Kanitz E., Weitzel J.M., Tuchscherer A., Stefaniak T., Jawor P., Wolffram S., Hammon H.M. (2016). Quercetin feeding in newborn dairy calves cannot compensate colostrum deprivation: Study on metabolic, antioxidative and inflammatory traits. PLoS ONE.

[10] Hwang E.-S., Kim G.-H. (2007). Biomarkers for oxidative stress status of DNA, lipids, and proteins in vitro and in vivo cancer research. Toxicology.

[11] Moolchandani A., Sareen M. (2018). A Review: Oxidative stress during lactation in dairy cattle. J. Dairy Vet. Sci..

[12] LeBlanc S., Lissemore K., Kelton D., Duffield T., Leslie K. (2006). Major advances in disease prevention in dairy cattle. J. Dairy Sci..

[13] Celi P. (2010). The role of oxidative stress in small ruminants’ health and production. Rev. Bras. Zootecn..

[14] McCoard S., Adiletta A., Jenkinson C., Peterson S., Kenyon P., Blair H. (2018). Brief communication: Impact of maternal plane of nutrition, ewe weight and twinning on fetal mammary gland development in sheep. N. Z. J. Anim. Sci..

[15] Reintke J., Brügemann K., Wagner H., Engel P., Wehrend A., König S. (2020). Phenotypic relationships between maternal energy metabolism and lamb body weight development during lactation for pure-and crossbred sheep populations in low and high input production systems. Small Rumin. Res..

[16] Lugarà R., Greve S., Sinz S., Marquardt S., Giller K., Chizzottin M.L. (2019). Effects of maternal and direct polyphenol intake on hepatic gene expression in lambs and kids. Energy and Protein Metabolism and Nutrition.

[17] Liguori G., Gentile C., Gaglio R., Perrone A., Guarcello R., Francesca N., Fretto S., Inglese P., Settanni L. (2020). Effect of addition of *Opuntia ficus-indica* mucilage on the biological leavening, physical, nutritional, antioxidant and sensory aspects of bread. J. Biosci. Bioeng..

[18] Amer F., Mobaraz S., Basyony M., Mahrose K., El-Medany S. (2019). Effect of using prickly pear and its by-products as alternative feed resources on performance of growing rabbit. Egypt. J. Rabbit Sci..

[19] Yahia E.M., Sáenz C. (2017). Cactus pear fruit and cladodes. Fruit and Vegetable Phytochemicals; Chemistry and Human Health.

[20] Díaz M.D.S.S., de La Rosa A.-P.B., Héliès-Toussaint C., Guéraud F., Nègre-Salvayre A. (2017). *Opuntia* spp.: Characterization and benefits in chronic diseases. Oxidat. Med. Cell. Longev..

[21] Cardador-Martínez A., Jiménez-Martínez C., Sandoval G. (2011). Revalorization of cactus pear (*Opuntia* spp.) wastes as a source of antioxidants. Food Sci. Technol..

[22] Zenteno-Ramirez G., Juárez-Flores B., Aguirre-Rivera J., Monreal-Montes M., García J.M., Serratosa M.P., Santos M.V., Pérez M.O., Rendon-Huerta J. (2018). Juices of prickly pear fruits (*Opuntia* spp.) as functional foods. Ital. J. Food Sci..

[23] Tahir H.E., Xiaobo Z., Komla M.G., Mariod A.A. (2019). Nopal cactus (*Opuntia ficus-indica* (L.) Mill) as a source of bioactive compounds. Wild Fruits: Composition, Nutritional Value and Products.

[24] El-Mostafa K., El Kharrassi Y., Badreddine A., Andreoletti P., Vamecq J., El Kebbaj M., Latruffe N., Lizard G., Nasser B., Cherkaoui-Malki M. (2014). Nopal cactus (*Opuntia ficus-indica*) as a source of bioactive compounds for nutrition, health and disease. Molecules.

[25] Costa R.G., Treviño I.H., Medeiros G.R.d., Medeiros A.N.d., Gonzaga Neto S., Azevedo P.S.d., Pinto T.F. (2013). Feeding behavior and performance of sheep fed cactus pear in substitution of corn. Rev. Bras. Zootecn..

[26] Elghandour M.M., Rodríguez-Ocampo I., Parra-Garcia A., Salem A.Z., Greiner R., Marquez-Molina O., Barros-Rodriguez M., Barbabosa-Pilego A. (2018). Biogas production from prickly pear cactus containing diets supplemented with *Moringa oleifera* leaf extract for a cleaner environmental livestock production. J. Clean. Prod..

[27] Makkar H.P. (2003). Quantification of Tannins in Tree and Shrub Foliage: A Laboratory Manual.

[28] Buchanan K., Burt de Perera T., Carere C., Carter T., Hailey A., Hubrecht R., Jennings D., Metcalfe N., Pitcher T., Peron F. (2012). Guidelines for the treatment of animals in behavioural research and teaching. Anim. Behav..

[29] Abou-Zeina H.A., Nasr S.M., Nassar S.A., Farag T.K., El-Bayoumy M.K., Ata E.B., Hassan N.M., Abdel-Aziem S.H. (2019). Beneficial effects of antioxidants in improving health conditions of sheep infected with foot-and-mouth disease. Trop. Anim. Health Prod..

[30] Gaines W.L., Davidson F.A. (1923). Relation between Percentage Fat Content and Yield of Milk: Correction of Milk Yield for Fat Content.

[31] NRC (2007). Nutrient Requirements of Small Ruminants: Sheep, Goats, Cervids, and New World Camelids.

[32] Van Kampen E., Zijlstra W. (1983). Spectrophotometry of hemoglobin and hemoglobin derivatives. Adv. Clin. Chem..

[33] Warnick G., Benderson J., Albers J. (1982). Dextran sulfate-Mg^2+^ precipitation procedure for quantitation of high-density-lipoprotein cholesterol. Clin. Chem..

[34] Nishikimi M., Roa N., Yogi K. (1972). Measurement of superoxide dismutase. Biochem. Biophys. Res. Commun..

[35] Aebi H. (1984). [13] Catalase *in vitro*. Methods Enzymol..

[36] Paglia D.E., Valentine W.N. (1967). Studies on the quantitative and qualitative characterization of erythrocyte glutathione peroxidase. J. Lab. Clin. Med..

[37] Tappel A.L., Zalkin H. (1959). Inhibition of lipid peroxidation in mitochondria by vitamin E. Arch. Biochem. Biophys..

[38] Chernow B., Alexander H.R., Smallridge R.C., Thompson W.R., Cook D., Beardsley D., Fink M.P., Lake C.R., Fletcher J.R. (1987). Hormonal responses to graded surgical stress. Arch. Intern. Med..

[39] NRC (2001). Nutrient Requirements of Dairy Cattle: 2001.

[40] AOAC (2006). Association of Official Analytical Chemists. Official Methods of Analysis.

[41] Van Soest P.v., Robertson J., Lewis B. (1991). Methods for dietary fiber, neutral detergent fiber, and nonstarch polysaccharides in relation to animal nutrition. J. Dairy Sci..

[42] Goering H.K., Van Soest P.J. (1970). Forage Fiber Analyses: Apparatus, Reagents, Procedures, and Some Applications.

[43] Van Soest P.J. (1973). Collaborative study of acid-detergent fiber and lignin. J. Assoc. Off. Anal. Chem..

[44] Ramos-Morales E., Arco-Pérez A., Martín-García A.I., Yáñez-Ruiz D.R., Frutos P., Hervás G. (2014). Use of stomach tubing as an alternative to rumen cannulation to study ruminal fermentation and microbiota in sheep and goats. Anim. Feed Sci. Technol..

[45] Konitzer K., Voigt S. (1963). Direct determination of ammonium in blood and tissue extracts by means of the phenol by chlorite reaction. Clin. Chim. Acta.

[46] Abo-Zeid H., El-Zaiat H., Morsy A., Attia M., Abaza M., Sallam S. (2017). Effects of replacing dietary maize grains with increasing levels of sugar beet pulp on rumen fermentation constituents and performance of growing buffalo calves. Anim. Feed Sci. Technol..

[47] Duncan D.B. (1955). Multiple range and multiple F tests. Biometrics.

[48] Chiba L.I. (2009). Animal Nutrition Handbook.

[49] Mills K.E., Weary D.M., von Keyserlingk M.A. (2020). Identifying barriers to successful dairy cow transition management. J. Dairy Sci..

[50] Winkler A., Gessner D.K., Koch C., Romberg F.-J., Dusel G., Herzog E., Most E., Eder K. (2015). Effects of a plant product consisting of green tea and curcuma extract on milk production and the expression of hepatic genes involved in endoplasmic stress response and inflammation in dairy cows. Arch. Anim. Nutr..

[51] Harding F. (1995). World milk production. Milk Quality.

[52] Shoorideh Z., Azadbakht M., Zarifkar A., Jafari A., Hossieni S. (2007). The effect of “*Vitex Agnus Castus*” folio extract on serum prolactin consentration of female rats in gastation. Iran. J. Biol..

[53] Svennersten-Sjaunja K., Olsson K. (2005). Endocrinology of milk production. Domest. Anim. Endocrinol..

[54] Gessner D., Koch C., Romberg F.-J., Winkler A., Dusel G., Herzog E., Most E., Eder K. (2015). The effect of grape seed and grape marc meal extract on milk performance and the expression of genes of endoplasmic reticulum stress and inflammation in the liver of dairy cows in early lactation. J. Dairy Sci..

[55] Silva E.T.D.S., Melo A.A.S.D., Ferreira M.D.A., Oliveira J.C.V.D., Santos D.C.D., Silva R.C., Inácio J.G. (2017). Acceptability by Girolando heifers and nutritional value of erect prickly pear stored for different periods. Pesqui. Agropecu. Bras..

[56] Adejoro F.A., Hassen A., Akanmu A.M. (2019). Effect of lipid-encapsulated acacia tannin extract on feed intake, nutrient digestibility and methane emission in sheep. Animals.

[57] Reda T.H., Atsbha M.K. (2019). Nutritional composition, antinutritional factors, antioxidant activities, functional properties, and sensory evaluation of cactus pear (*Opuntia ficus-indica*) seeds grown in tigray region, Ethiopia. Int. J. Food Sci..

[58] Catunda K.L.M., de Aguiar E.M., de Góes Neto P.E., da Silva J.G.M., Moreira J.A., do Nascimento Rangel A.H., de Lima Júnior D.M. (2016). Gross composition, fatty acid profile and sensory characteristics of Saanen goat milk fed with *Cacti varieties*. Trop. Anim. Health Prod..

[59] Aguiar S., Cottica S., Boeing J., Samensari R., Santos G., Visentainer J., Zeoula L. (2014). Effect of feeding phenolic compounds from propolis extracts to dairy cows on milk production, milk fatty acid composition, and the antioxidant capacity of milk. Anim. Feed Sci. Technol..

[60] Vasta V., Daghio M., Cappucci A., Buccioni A., Serra A., Viti C., Mele M. (2019). Invited review: Plant polyphenols and rumen microbiota responsible for fatty acid biohydrogenation, fiber digestion, and methane emission: Experimental evidence and methodological approaches. J. Dairy Sci..

[61] Toral P.G., Monahan F.J., Hervás G., Frutos P., Moloney A. (2018). Review: Modulating ruminal lipid metabolism to improve the fatty acid composition of meat and milk. Challenges and opportunities. Animal.

[62] Urrutia N.L., Harvatine K.J. (2017). Acetate dose-dependently stimulates milk fat synthesis in lactating dairy cows. J. Nutr..

[63] Borges L.D.A., Júnior V.R.R., Monção F.P., Soares C., Ruas J.R.M., e Silva F.V., Rigueira J.P.S., Costa N.M., Oliveira L.L.S., de Oliveira Rabelo W. (2019). Nutritional and productive parameters of Holstein/Zebu cows fed diets containing cactus pear. Asian Australas J. Anim. Sci..

[64] Toral P.G., Hervás G., Bichi E., Belenguer Á., Frutos P. (2011). Tannins as feed additives to modulate ruminal biohydrogenation: Effects on animal performance, milk fatty acid composition and ruminal fermentation in dairy ewes fed a diet containing sunflower oil. Anim. Feed Sci. Technol..

[65] Natalello A., Hervás G., Toral P.G., Luciano G., Valenti B., Mendoza A.G., Pauselli M., Priolo A., Frutos P. (2020). Bioactive compounds from pomegranate by-products increase the in vitro ruminal accumulation of potentially health promoting fatty acids. Anim. Feed Sci. Technol..

[66] Gowane G., Chopra A., Prakash V., Prince L. (2014). The role of maternal effects in sheep breeding: A review. Indian J. Small Rumin..

[67] Cuevas Reyes V., Santiago Hernandez F., Flores Najera M.D.J., Vazquez Garcia J.M., Urrutia Morales J., Hosseini-Ghaffari M., Chay-Canul A., Meza-Herrera C.A., Gonzalez-Bulnes A., Martin G.B. (2020). Intake of spineless cladodes of *Opuntia ficus*-Indica during late pregnancy improves progeny performance in underfed sheep. Animals.

[68] David I., Bouvier F., Ricard E., Ruesche J., Weisbecker J.-L. (2013). No direct by maternal effects interaction detected for pre-weaning growth in Romane sheep using a reaction norm model. Genet. Sel. Evol..

[69] Dhaoui A., Chniter M., Atigui M., Dbara M., Seddik M.-M., Hammadi M. (2019). Factors affecting the milk yield and composition over lactation of prolific SD’man ewes in Tunisian oases. Trop. Anim. Health Prod..

[70] Saxena M., Saxena J., Nema R., Singh D., Gupta A. (2013). Phytochemistry of medicinal plants. J. Pharmacogn. Phytochem..

[71] Lim K.-T., Lee J.-C. (2000). Effects of cactus and ginger extracts as dietary antioxidants on reactive oxidant and plasma lipid level. Food Sci. Biotechnol..

[72] Louacini B., Dellal A., Halbouche M., Ghazi K. (2012). Effect of incorporation of the spineless *Opuntia ficus-indica* in diets on biochemical parameters and its impact on the average weight of ewes during the maintenance. Glob. Vet..

[73] Zhu R., Hou Y., Sun Y., Li T., Fan J., Chen G., Wei J. (2017). Pectin penta-oligogalacturonide suppresses intestinal bile acids absorption and downregulates the FXR-FGF15 axis in high-cholesterol fed mice. Lipids.

[74] Osuna-Martínez U., Reyes-Esparza J., Rodríguez-Fragoso L. (2014). Cactus (*Opuntia ficus-indica*): A review on its antioxidants properties and potential pharmacological use in chronic diseases. Nat. Prod. Chem. Res..

[75] Oh P.-S., Lim K.-T. (2006). Glycoprotein (90 kDa) isolated from *Opuntia ficus-indica* var. *saboten* MAKINO lowers plasma lipid level through scavenging of intracellular radicals in Triton WR-1339-induced mice. Bioll. Pharm. Bull..

[76] El-Neney B.A., Zeedan K.I., El-Kotamy E., Gad G.G., Abdou A. (2019). Effect of using prickly pear as a source of dietary feedstuffs on productive performance, physiological traits and immune response of rabbit. 2-prickly pear peels. Egypt. J. Nutr. Feed..

[77] Vakili A., Khorrami B., Mesgaran M.D., Parand E. (2013). The effects of thyme and cinnamon essential oils on performance, rumen fermentation and blood metabolites in Holstein calves consuming high concentrate diet. Asian Australas J. Anim. Sci..

[78] López-Romero P., Pichardo-Ontiveros E., Avila-Nava A., Vázquez-Manjarrez N., Tovar A.R., Pedraza-Chaverri J., Torres N. (2014). The effect of nopal (*Opuntia ficus indica*) on postprandial blood glucose, incretins, and antioxidant activity in Mexican patients with type 2 diabetes after consumption of two different composition breakfasts. J. Acad. Nutr. Diet..

[79] Gessner D., Ringseis R., Eder K. (2017). Potential of plant polyphenols to combat oxidative stress and inflammatory processes in farm animals. J. Anim. Physiol. Anim. Nutr..

[80] Hilario M.C., Puga C.D., Ocana A.N., Romo F.P.-G. (2010). Antioxidant activity, bioactive polyphenols in Mexican goats’ milk cheeses on summer grazing. J. Dairy Res..

[81] Siqueira M.C., Ferreira M.D.A., Monnerat J.P.I.D.S., Silva J.D.L., Costa C.T., da Conceição M.G., de Andrade R.D.P., Barros L.J., Melo T.T.D.B. (2017). Optimizing the use of spineless cactus in the diets of cattle: Total and partial digestibility, fiber dynamics and ruminal parameters. Anim. Feed Sci. Technol..

[82] Lopes L.A., Ferreira M.d.A., Batista Â.M.V., Maciel M.d.V., Barbosa R.d.A., Munhame J.A., Silva T.G.P.d., Cardoso D.B., Véras A.S.C., de Carvalho F.F.R. (2020). Intake, digestibility, and performance of lambs fed spineless cactus cv. Orelha de Elefante Mexicana. Asian-Australas J. Anim. Sci..

[83] Inácio J.G., Ferreira M.d.A., da Conceição M.G., dos Santos D.C., de Oliveira J.C., Chagas J.C.C., Moraes G.S.d.O., Silva E.T.d.S. (2019). Nutritional and performance viability of cactus *Opuntia*-based diets added to concentrate levels for Girolando lactating dairy cows. Asian-Australas J. Anim. Sci..

[84] Costa R.G., Beltrão Filho E.M., de Medeiros A.N., Givisiez P.E.N., do Egypto R.d.C.R., Melo A.A.S. (2009). Effects of increasing levels of cactus pear (*Opuntia ficus-indica L. Miller*) in the diet of dairy goats and its contribution as a source of water. Small Rumin. Res..

[85] Misra A., Mishra A., Tripathi M., Chaturvedi O., Vaithiyanathan S., Prasad R., Jakhmola R. (2006). Intake, digestion and microbial protein synthesis in sheep on hay supplemented with prickly pear cactus [*Opuntia ficus-indica* (L.) Mill.] with or without groundnut meal. Small Rumin. Res..

[86] Ben Salem H., Nefzaoui A., Abdouli H., Ørskov E. (1996). Effect of increasing level of spineless cactus (*Opuntia ficus-indica* var. *inermis*) on intake and digestion by sheep given straw-based diets. Anim. Sci..

[87] Morsy T., Kholif S., Kholif A., Matloup O., Salem A., Elella A.A. (2015). Influence of sunflower whole seeds or oil on ruminal fermentation, milk production, composition, and fatty acid profile in lactating goats. Asian Australas J. Anim. Sci..

[88] Kholif A., Khattab H., El-Shewy A., Salem A., Kholif A., El-Sayed M., Gado H., Mariezcurrena M. (2014). Nutrient digestibility, ruminal fermentation activities, serum parameters and milk production and composition of lactating goats fed diets containing rice straw treated with Pleurotus ostreatus. Asian Australas J. Anim. Sci..

